# Pharmacological management of visual hallucinations in dementia with Lewy body – A case presentation

**DOI:** 10.1192/j.eurpsy.2023.1993

**Published:** 2023-07-19

**Authors:** P. Tirlea, R. Tipa, L. Ignat

**Affiliations:** 1 Alexandru Obregia Clinical Psychiatry Hospital; 2 Carol Davila University of Medicine and Pharmacy, Bucharest, Romania

## Abstract

**Introduction:**

Dementia with Lewy bodies(DLB) and Parkinson’s disease dementia(PDD) make up for about 20% of dementia cases, with a significant overlap of clinical features. They are described as separate entities in the DSM-5 with an arbitrary delimitation based on the onset of cognitive decline in relation to parkinsonism.Visual hallucinations are a common clinical feature. Treatment consists of low dose antipsychotics, generally quetiapine or clozapine being used.

**Objectives:**

Case presentation and reflection on pharmacological treatment

**Methods:**

Review of the clinical file of a patient with DLB

**Results:**

A 76 year old female was referred to our clinic with a recent history of complex visual hallucinations and delusional thoughts. The onset of parkinsonism was made 8 months prior to admission and treatment with IMAO-B Rasagiline and a combination of Levodopa was initiated. The patient had no psychiatric hospitalization history. Her comorbidities include hypertension, dyslipidemia and osteoporosis, for which she received specific treatment. The onset of complex visual hallucinations was one month prior to admission. A trial with small dose clozapine was initiated in an out-patient setting and dropped out due to intolerance.

During the hospitalization she was describing recurrent complex visual hallucinations in the form of people engaging in sexual activities in front of her and suspected her husband of involvement in these acts. She was also experiencing tactile and proprioceptive hallucinations, interpreting them as harmful laser beams resulting in skin marks (age spots were present). Sun downing syndrome was present, consisting of fluctuating cognition, worsening of temporo-spatial orientation, marked anxiety as the visual hallucinations became more vivid. CT scan showed moderate atrophy and psychological testing indicated moderate cognitive decline.

Treatment with Rasagiline was interrupted as it can worsen psychotic features, by raising dopamine levels. Levodopa was reduced to the minimum efficient dose for parkinsonism as it can cause agitation and worsening of visual hallucinations. Treatment with small dose quetiapine (100 mg per day) was initiated, the patient experiencing severe hypotension due to neuroleptic sensitivity. Quetiapine was continued for about 3 months, with the aggravation of the visual hallucinations and encapsulated delusional thinking. Small dose Clozapine (25 mg per day) was rechallenged, with favorable outcome. Some visual disturbances were still present but less bothersome. Lorazepam was used for the management of insomnia and psychomotor agitation and the cholinesterase inhibitor Rivastigmine for managing the behavioral symptoms and cognitive decline.

**Conclusions:**

Visual hallucinations are often a bothersome clinical feature in DLB. Treatment and diagnosis is often challenging. Clozapine is a good option for managing visual hallucinations in DLB.

**Disclosure of Interest:**

None Declared

